# Apple orchard waste recycling and valorization of valuable product-A review

**DOI:** 10.1080/21655979.2021.1872905

**Published:** 2021-01-20

**Authors:** Yumin Duan, Sanjeet Mehariya, Aman Kumar, Ekta Singh, Jianfeng Yang, Sunil Kumar, Huike Li, Mukesh Kumar Awasthi

**Affiliations:** a College of Natural Resources and Environment, Northwest A&F University, Yangling, Shaanxi ProvinceChina; b Department of Engineering, University of Campania “Luigi Vanvitelli”, Aversa (CE), Italy; c CSIR-National Environmental Engineering Research Institute, NagpurMaharashtra, India; d Swedish Centre for Resource Recovery, University of Borås, Borås, Sweden

**Keywords:** Apple orchard waste, waste management, value-added products, resource recovery

## Abstract

Huge quantities of apple orchard waste (AOW) generated could be regarded as a promising alternative energy source for fuel and material production. Conventional and traditional processes for disposal of these wastes are neither economical nor environment friendly. Hence, sustainable technologies are required to be developed to solve this long-term existence and continuous growing problem. In light of these issues, this review pays attention towards sustainable and renewable systems, various value-added products from an economic and environmental perspective. Refined bio-product derived from AOW contributes to resource and energy demand comprising of biomethane, bioethanol, biofuels, bio-fertilizers, biochar, and biochemicals, such as organic acid, and enzymes. However, the market implementation of biological recovery requires reliable process technology integrated with an eco-friendly and economic production chain, classified management.

## 1. Introduction

**Figure 1.**
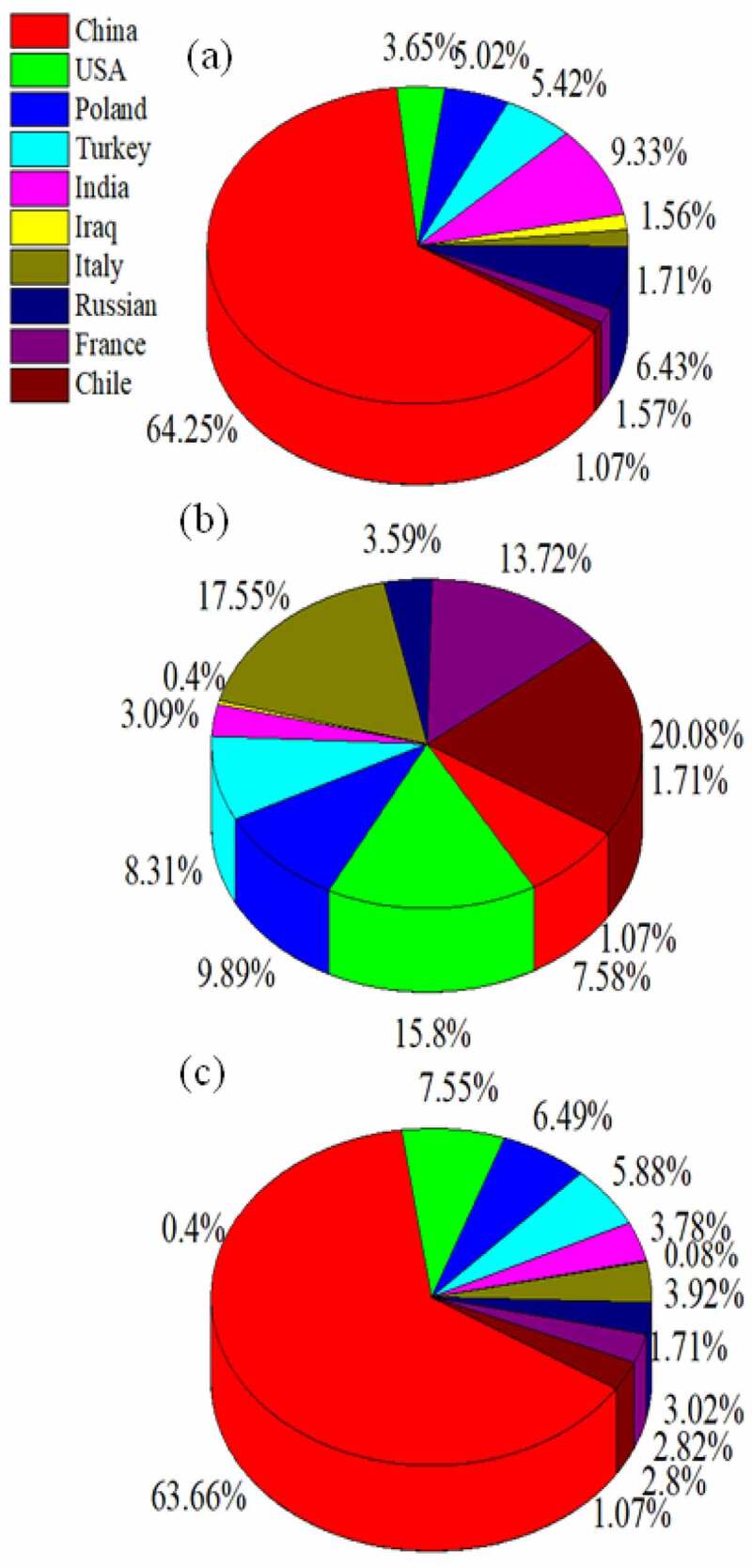
The apple (a) Yield (b) Production and (c) Area harvested percentage of the top ten countries in the world [7]

According to the statistics of Food and Agriculture Organization, the global apple area harvested and production approximately reached 4.9 million hectares and 86.14 million tons, respectively, in 2018 and the top 10 countries are presented in Table 1 and Figure 1. Notably China ranks first in area harvested (11.87 million hectares) and production (39.23 million tons), followed by the United States and Poland with 4.65 million and 3.99 million tons of apple production [1]. Interestingly, apple orchard has generated a huge quantity of blossom, pruning branches, fruit thinning and trunks during the growth of apple trees, harvesting of apples, and recycling of this biomass residue is major problems for local farmers [2]. Generally, recycling of apple orchard residues need many practical requirement and approaches, such as on-site disposal by open combustion, mulching directly and landfilling, which bring many inevitable series of ecological risk and social drawbacks. In addition, it helps microbes increase the risk of diseases and decrease available nitrogen in the soil and then further un-balance C/N ratio. This increased the investment cost owing to the required extra fertilizer application to maintain fruit yield and quality [3,4]. However, in the current situation woody component and apple residues are considered as waste instead of resource. In fact, orchard residues are part of the abundant biomass that could be known as the main alternative source of renewable energy and can produce multi-benefit products [5,6].

**Table 1.** The apple area harvested and production amounted to the top 10 countries in the world [7]

Country	Production (hg/ha)	Area harvested (tonnes)
China	189,388	39,235,019
United States of America	394,802	4,652,500
Poland	247,205	3,999,523
Turkey	207,565	3,625,960
India	77,309	2,327,000
Iraq	9922	49,813
Italy	438,574	2,414,921
Russian Federation	89,704	1,859,400
France	342,935	1,737,412
Chile	501,715	1,727,277

Thus, recycling of apple orchard residues and conversion of energy is one of the best sustainable alternative approach and circular economy as an imperative trend. However, extensive utilization of fossil resources may lead to its exhaustion due to the non-renewability, and at the same time also bring environmental pollution such as the emissions of coal and gas-diesel oil found to be 46 thousand and 48 thousand gigagrams (CO_2_eq) in China [7,]. Reports by the International Energy Agency estimated that the world energy consumption is increased by 48% from 2012 to 2040 [8]. According to global attention, renewable energy accounted for 19% of the global energy consumption in 2012 and would contribute 55–75% by 2050 in the European Union [7]. Kumar et al. [9] pointed out that the biomass would contribute 15–50% in world primary energy consumption until 2050. Consistently, Alavijeh and Yaghmaei [7] reported biomass as the main contributor of renewable energy and the stored energy from global biomass annually increasing by approximately 8 times than total global energy demand. Therefore, the development and economic utilization of biomass contributing to energy demand is an environment potential friendly approach that will be attracting more and more attention across the globe.

Apple orchard residues emerged as a promising source for diverse value-added products derived from cellulose-rich materials (branches and leaves) that can be converted into various biofuels such as hydrogen, ethanol, and methane through various biorefinery approaches [10]. In addition, along with the increasing demand for food diversity and chemicals requirement, the safety of food and biochemical are essential concerns of manufacturers and consumers. In case of nutritional value, various carbohydrate-rich biomass includes apple pomace as suitable material for biofertilizer and biochemical production. In addition, it helps to increase the total relative abundance of microbes including their enzymes, organic acids, pectin, polyphenols, biopolymers, and other compounds. Finally, these microbes certainly help to increase the nutrient level and chemical recovery [11].

The conversion of biomass into bio-energy, nutrient, and biochemical involve the basic steps of collection, transportation, pretreatment, and processing. Apple pomace residues are complex to dispose of owing to high moisture content and organic compound susceptible that is prone to enzymatic degradation and rapid oxidation. The fiber-rich residue is constrained by pretreatment and utilized efficiency due to its hard decomposed complex structure [5]. Additionally, various obstacles such as insufficient social awareness, unfavorable site, or logistical conditions like logistic costs, raised investment, and complicated process cause difficulty of getting sustained financial support. The uncertainty of seasonal and regional variations, infrastructure technical barriers, and the aforementioned biomass properties restricted the feasibility of widespread commercialization in the global market. Based on present status, this article comprehensively describes the available and potential value-added products and discusses the current challenges and future development prospects. It also proposed the availability of apple orchard biomass residues for bio-energy, nutrient, and chemicals recovery, aiming to motivate public members, commerce and industry departments to enhance awareness of participation in apple orchard recycle management.

## 2. Resource recovery from apple orchard waste

Apple orchard waste recycling needs a sustainable and environmentally friendly manner. The optimal practice approach for sustainable orcharding not only includes minimization of waste biomass but also conservation of waste into useful multi-product, energy, and also help in the circular economy. As reported in a chemical composition study [12], AOW is an important bio-resource, pruned leaves, twigs, stems, and branches along with immature fruits all are rich in phenol (the highest total phenolic content) was shown by leaves, stems, and immature fruits with 810.2, 320.2–245.0, and 324.4 mg/100 g dry weight, respectively. Although the ripe apple fruits showed the lowest content, i.e., 42.7 mg/100 dry weight of phenol. Waste pruning is found to be rich in polyphenols. It suggested that the possible application of AOW (mainly pruning) is a potential source to produce polyphenols that has high value due to their antioxidant properties with respect to human health. So it is proposed that these compounds might be used as natural antioxidants in the food industry and supplement artificial antioxidants, and thereby add an alternate and renewable source. It can also be used as great potential for the processing of biofuels such as methane, bioethanol, butanol, and hydrogen. Additionally, apple waste is as a green energy or alterations in the form of biofertilizer that can be applied directly to the soil [13]. Major applications of AOW have been dealt with as follows:

### 2.1. Biofuels recovery

For growing the energy supply protection and decreasing emissions of greenhouse gas, both by rising and expanding dependency on fossil fuels, thus, the replacement of fossil fuels by renewable energy sources (RES) are one of the key issues [14]. Bioenergy is one of the promising, inexhaustible, and renewable sources of energy that help tackle increasing ecological, economic, and technical challenges looking toward the decline of fossil fuels. The composition and characteristics of feedstock are the most significant part of sustainable growth and supply of bioenergy. Among the various substrates, waste production has received extraordinary acceptance for the maintenance of environmental uprightness.

A high energy potential is obtained from several residues of industrial and agricultural activities describes biomass as a renewable energy. Generally, the waste biomass’s higher heating value (HHV) lies between 17 and 21 MJ per kg [15]. Apple orchards pruned wood is becoming one of the most valued biofuels with 13.6–14.6 MJ/kg of calorific value (as high as brown coal), less hazardous gaseous emissions, and low ash content. Pruned biomass’s theoretical capacity from permanent crops in the EU28 is estimated to be approx. 246 PJs per year and AOW account for 4.2% of the permanent crop area [16,17]. Pruning-to-energy (PtE) may be particularly significant in countryside areas categorized by a huge percentage of apple orchards and restricted access to forest resources in the region. Unluckily, technology is not being commercialized and most of this potential is being unused.

**Figure 2.**
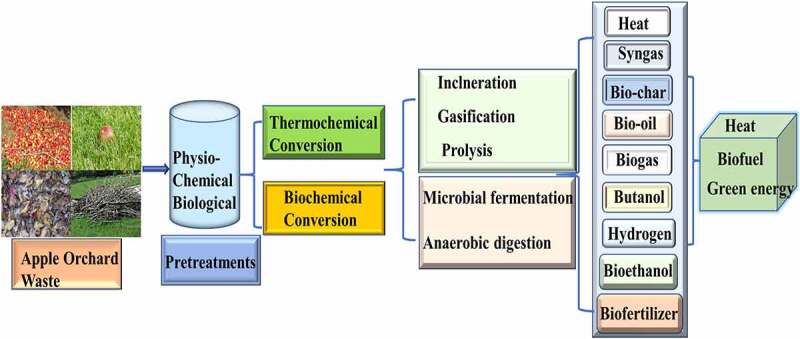
Resource recovery processes from apple orchard waste

Apple orchard waste has also turned out to be a fairly cheaper source of ethanol production and the common people and the poor farmers will be benefitted greatly from this. The research conducted by used apple pomace and rotten banana as a substrate to produce ethanol by fermenting it with *Saccharomyces cerevisiae* [10]. Approximately 48% of the alcohol is obtained after distillation, and we may assume that a higher concentration of alcohol may be obtained after re-distillation of the substance. A higher concentration of alcohol can also be used as a biofuel. Conversion of waste to energy leads to the protection of resources and works in an environmental friendly manner on a sustainable basis. Figure 2 shows the resource recovery processes from AOW. General gaseous and liquid biofuels are explored in detail in the following segments.

#### 2.1.1. Biogas

Biogas is generally produced by anaerobic digestion and comprises of methane, carbon dioxide, hydrogen sulfide, and water vapor by 45–70%, 30–45%, 0.5–1.0%, and 1–5%, respectively, as well as less quantity of other gases (nitrogen, ammonia, hydrogen, etc.) [18]. The product formed during the entire process differs from the related microbial population type as microbes control the three stages of anaerobic digestion (methanogenesis, acidogenesis, and hydrolysis). The single-stage digestion of apple fruit waste is favored because of its simple design and lesser investment costs. However, the activity and growth of methanogens are affected by the high loading rate which is the main difficulty associated with single-phase digestion. The metabolic behavior of acidogenic microbes rises at high loading levels causing increased hydrogen concentration, whereas methanogenic organisms cannot raise the behavior and this discrepancy brings about the end of methane generation. The high organic biodegradability of apple waste induces broad volatile fatty acids and a rapid acidification that inhibits the function of methanogenic microorganisms. While production of methane is a primitive method but still faces a big challenge to improve the efficiency and performance of gas production.

By eliminating most of the hydrogen sulfide and CO_2_ from the biogas, the key objective can be achieved by obtaining high CH_4_ content in biogas. The process of amine scrubbing is the technique primarily employed for biogas application. Hydrogen sulfide and water is added in the first step and then using a stimulated carbon filter, desulfurization is done. The loss of biomass in systems is another vital challenge with a high rate of hydraulic charge, which has been overcome by developing reactors with excessive loading levels and short hydraulic retention time (HRT). The homogeneity of particle size is lacked by digested slurries in the single-stage digestion process, leading to the layers or phases of different densities formation in the reactor. Generally, it inhibits the appropriate mixing of hampers and propellers and then it is beneficial to periodically remove the layers in the reactor. In the batch method, organic waste is usually fed once in the reactor where it goes through consecutive decomposition. It has not been able to achieve considerable market share by the batch digester because of its HRT [19]. Digestion of vegetable/fruit waste through semi-continuous or continuous anaerobic digestion is one of the most prevalent and remarkable approaches [20]. More flexible bioreactors are required to digest vegetable/fruit waste in one phase with continuous-stirred tank reactors (CSTR) [21]. Li et al. [22] investigated apple pulp being used to promote buffer capacity and nutrient balance improving the stability of manure anaerobic digestion system. The highest methane production reached 0.34 L g^−1^ VS_added_, co-digest of apple pulp, cow slurry, and olive pomace have produced stable biogas of 400 L/kg volatile solids. Jansson et al. [23] investigated CSTR for digestion of residue of vegetables, the digestion of sugar beet pulp and asparagus waste led to a methane generation of 0.263 and 0.230 l g^−1^ VS fed, respectively, and reduction in VS equal to 95.2% and 89.7%. In comparison to single-phase digestion, batch sequencing reactors are becoming more critical for anaerobic digestion, when it comes to enhancing solid retention and unsettlement. Kafle et al. [24] performed batch and continuous anaerobic digestion to produce biogas from apple waste and swine manure, the final methane, and biogas productivity from apple waste detected was 252 and 510 mL/g total chemical oxygen demand (TCOD). For anaerobic treatment, the usage of two-stage bioreactors is very significant because of its HRT flexibility, its organic load rates due to the sequential arrangement of different bioreactors for acidogenesis and methanogenesis [21]. For vegetable and fruit waste, the effectiveness of a sequence anaerobic batch reactor is shown to be good and used in two-phase digestion by Bouallagui et al. [19]. It resulted in substantial development of biogas with improved effluent capacity, reduced chemical oxygen demand by 96%. Xiao et al. [25] digested tomato waste with cattle dung in an anaerobic reactor which also resulted in an increase from 0.33 to 0.700 dm^3^ d^−1^ of biogas. Anaerobic treatment of fruit/vegetable waste is rich in organic matter and is beneficial as it produces more methane along with slurry which can be applied as a soil conditioner.

#### 2.1.2. Biodiesel

Low aliphatic alcohols and high fatty acid alkyl esters are constituents of biodiesel. As fatty acid depends on calorific value, its composition of triglycerides in the feedstock decides their utility. The presence of non-sponifiables, impurities, and moisture are other chemical and physical parameters that regulate feedstock efficiency [26]. Biodiesel cost frequently makes up around 70–95% of the operational costs which is defined by the selection of feedstock [27]. Vegetable oil is environmentally friendly, readily available, and naturally sustainable, therefore for biodiesel production, it is one of the productive and effective substrates. Both non-edible and edible oils were broadly employed for this purpose. Non-nutritious oils suitable for biodiesel use include *Ricinus communis, Hevea brasiliensis, Nicotiana tabacum, Jatropha curcus, Madhuca longifolia, Attalea speciosa*, and *Pongamia glabra* to name but a few [28]. Using *Eruca sativa* Gars oil for production of biodiesel, and resulting in a conversion rate of 98% [29].

Su et al. [30] stated that the biodiesel can be generated using high-temperature pretreated kitchen garbage. Pizarro et al. [31] used waste (bleaching earth) for the production of 55% biodiesel throughout the process of refining crude vegetable oil. Either by using or without using a catalyst, biodiesel production is also attained through vegetable oils trans-esterification with simple alcohols. The parameters affecting biodiesel production are purity of reactants, mixing velocity, alcohol to oil ratio, and reaction temperature [2]. The optimization of these factors has been extensively studied to enhance the yield of biodiesel. For trans-esterification, alkaline and acidic catalysts have been widely used to increase the biodiesel production. Using NaOH as an alkaline catalyst, recorded the greatest biodiesel yield for the transformation of cotton oil and soybean oil and catalysts for the production of biodiesel using soap stocks for both base (NaOH) and acid (H_2_SO_4_) [32]. Recently, in the production of biodiesel, enzymes, and solid catalyst are used. In a similar study, using a carbon-based solid acid catalyst for oleic acid supplemented cotton oil trans-esterification (50% w/w), and recorded 80.5% triglyceride conversion [33]. Biodiesel synthesis based on enzymes requires the lipase usage which offers an additional advantage in the purification and simple separation of the reaction products. At Lvming Co. Ltd. in China, immobilized lipase is used for trans-esterification of waste cooking oil to produce further enzymatic development of methyl ester fatty acid [28]. Aguilar et al. [34] recorded yields of up to 30% using entire cell *Rhizopus oryzae IFO 4697* as biocatalysts. Several methods have been employed for the biodiesel production, aiming at the conservation of material and energy. In one such effort, with the taking on ultrasonic irradiation (20 kHz) for Canola oil trans-esterification, approximately 99% yield was achieved in 50 min [35]. Lee et al. [36] used supercritical fluid extraction for obtaining a high yield of biodiesel (approx. 100%) in 45 min of incubation time from waste canola oil. Research on the development of the trans-esterification cycle is underway with an emphasis on both yield and protection of the environment and the conservation of resource in the form of economy, recovery, and quality of the feedstock.

#### 2.1.3. Bioethanol and Biobutanol

Apple waste is a rich source of polysaccharides (lignin, hemicellulose, and cellulose) and may be introduced for butanol and ethanol production to solid-state fermentation. Further it is used for multiple purposes such as a supplement to liquid fuels and solvent in many industries [37]. The simple procedures for transforming starch and sugars from crops are commercially used and well known. Biomass fermentation, saccharification, and pretreatment are process which are used for biofuel production using AOW. For many years, ethanol has been a vital chemical in industry. In fact, in comparison to fossil fuels, ethanol is more environmentally friendly and is used as a substitute for gasoline. Micro-organisms of potential ethanol fermentation include recombinant *Escherichia coli, Zymomonas mobilis, Saccharomyces cerevisiae,* and *C. thermocellum* [38].

Various studies have confirmed the potential of apple pomace as a viable substrate for bioethanol production [38–39]. Dhillon et al. [40] demonstrated the efficient method for mitigation of waste disposal, the ethanol yield was 16.09% (v/w) with 0.15% (w/w) of apple pomace by fermentation, 0.08 million ton bioethanol have been produced from 0.4 million ton apple pomace waste in the US. Bioethanol production is reported by Zhang et al. using a 5-l scale production of bioethanol from various agricultural biomasses [41], mainly produced by Clostridia, require rod-shaped, anaerobic, and spore-forming gram-positive bacteria. It is very small but able to generate a huge quantity of butanol throughout fermentation. Ethanol, butanol, and acetone are produced in the ratio of 3:6:1 by Clostridia fermentation of the saccharified lignocellulosic [42]. Voget et al. [43] announced the development of 2.2% of the bio-butanol from apple pomace. Patakova et al. stated using molasses of sugar beet and maize to produce 13.70 and 15.23 g L–1 bio-butanol [44]. Bio-butanol and bioethanol are used as favorable substitutes to the transport fuels dependent on petroleum. The fruit waste is one of the significant sources of biomass with the ability to be transformed into butanol and ethanol. The conversion into biofuel from these wastes not only generate valuable end-products but also decreases the costs of waste disposal.

#### 2.1.4. Biohydrogen

Bio-hydrogen is considered as a potential biofuel due to ease of use, high energy content, and environmentally friendly nature. Applications of hydrogen energy are reliable with the high transformation efficiency attained from cells of fuel [45]. Because of zero CO_2_ emission it is one of the cleanest fuels and has a potential to substitute depleted oil reserves [46]. Production of fermentative hydrogen through dark and light-dependent fermentation processes has multiple advantages among all of the hydrogen production methods. Under anaerobic condition, dark fermentation occurs which increases the hydrogen production rate and therefore reduces costs of production from the light-dependent process. Substrates utilized during the production of bio-hydrogen with appropriate bio-process tools should be lack of nitrogen and solids rich in carbohydrate. Apple waste is considered a possible natural substrate with the potential to generate energy due to its easy biodegradability and high organic content amongst all waste. Other conditions for improving productivity are cultures-co-culture or mixed or pure. Potential microorganisms that contribute significantly to the development of bio-hydrogen are violet non-sulfur bacteria, *Cyanobacteria*, and green algae, which are fermentative, photosynthetic, etc [47]. Certain methods for raising the yield of H_2_ include temperature, pH, availability of nutrients, reduction in liquid phase H_2_ and CO_2_ levels, gas conservation, and pretreatment [48].

Such approaches are used by researchers around the world to increase the generation of bio-hydrogen. Mohanakrishna et al. [49] produced hydrogen using vegetable wastes and with sewage supplementation exhibited a rise of up to 55%. Similarly, Yao et al. [50] explored the hydrogen production from apple tree leaves by microwave pyrolysis, the higher heating value (HHV) of apple tree leaves is 18.80 MJ/kg, leaves biochar reached around 25.94 MJ/kg. Tenca et al. [51] adopted a different strategy for achieving maximum production of hydrogen without external control of the pH, they co-fermented vegetable waste using content-rich in alkali, i.e., pig dung, in order to circumvent changes of external pH. Lukitawesa et al. and Dong et al. used waste produced from potato processing industries to increase bio-hydrogen production to 55% from 15% [52]. Lu et al. [53] studied the possible of renewable energy bio-hydrogen product derived from rotten apples waste with photosynthetic bacteria, and obtained the maximum hydrogen yield was 111.85 ± 1 mL H_2_/g TS. Using fruit waste for bio-hydrogen production is an upright technique for transforming waste into energy and addressing energy security and environmental concerns. Treatment systems may be configured to extract energy from waste nutrients based on the initial screening and characterization of the waste. The use of microbes for the breakdown of discarded biomass of fruit into a preferred form of fuel is currently one of the supreme recognized approaches for the management of these wastes.

### 2.2. Nutrient recovery

Sustainable management of nutrition in orchard focusses on optimizing the usage of nutrient sources and the requirement for external inputs. Significant portions of the nutrients absorbed by orchard plants from the soil return to the soil each year through abscised pruning wood, leaves, and rhizodeposition biomass. Similarly, biomass mowed from grasses in orchard could also produce quantities of nutrients [54]. As a result of biomass decomposition, the increase of soil organic matter indirectly affects soil fertility and the availability of nutrients. Thus, improving the usage of nutrient resources residing efficiency in apple orchards are necessary to resolve productivity and environmental aspects [55]. Waste is earlier either burned or disposed of in landfills, resulting in very large quantities of greenhouse gases being released in the environment, while proper resource treatment can realize nutrient recovery and benefit soil quality. Several nutrient recovery processes are as follows:

#### 2.2.1. Composting

Composting is one of the best-known recovering methods to produce organic waste conditioners, which is the method of rotting organic material (food garbage, animal waste, crop residues, and municipal waste) by microbes under-regulated conditions. Apple orchard waste of leaves, grass, and branch are a good substrate to provide nitrogen and promote composting. Kopcic et al. verified the high degradation (53.1%) during apple and tobacco waste composting [56]. Composting offers a road map to sustainable disposal of organic waste materials for recovery of nutrients by providing a lesser risk of environmental liabilities. It turns a large portion of solid waste into a marketable commodity, the final product of compost is used as a conditioner to decompose recycled wastes and act as a source of nutrients. It is a well-cradle-to-cradle strategy in which organic matter return to earlier plant-taken soil. It helps to improve the soil quality, increase water retention capacity, availability of nutrient uptake, and contributes to sustaining the production of healthy crops.

Commonly, aerobic composting produces a more natural organic product that is primarily practiced in agricultural yields. Organically certified compost was reported to contain 2–2.5% of the N [56] that could provide the potential for nutrient recovery if properly managed. In addition, biochar made from apple branches or leaves used as an additive in composting demonstrates better efficiency aspect of increased temperature, pH, humification, and product quality, reduced composting duration, and greenhouse gases emission [57]. The composting method can be established using the following direction and chemical equation:

Organic waste (Protein + Cellulose + Lignin) + O_2_ → CO_2_ + H_2_O + Compost + Heat

#### 2.2.2. Vermicomposting

One potential method for treating rotting apple waste biomass is vermicomposting that makes use of earthworms to decompose into compost. It is the process of organic waste digestion performed by earthworms, accompanied by excretion through the metabolic system, during which their biological activities raise nutrient levels in organic waste [58]. Vermicompost contains more soluble levels of the nutrients and organic matter with the enhanced quality compared to conventional compost [59]. Hanc and Chadimova [60] evaluated the feasibility of vermicomposting-based apple pomace waste and stated that vermicomposting was an appropriate technique to decompose apple pomace waste by converting it into value-added products. Ultimate product value also enhanced with 2.8% N, 0.85% P, 2.3% K. García-Sánchez et al. [61] performed vermicomposting in the mixture of apple and grape pomace, digestate, and horse manure, the mature and stable end product for the agronomical application, concluded that apple pomace waste is a good feedstock and helps earthworm to convert into a value-added product.

It is an environmentally friendly and reasonable approach to be used due to the wide substrate of food, plants, animals, and sewage waste. Research shows the vermicomposting cycle is a good method to recycle organic waste and covert high-quality compost with greater bio-available nutrients [61]. It is a mechanism in which earthworms generate useful dung (vermicompost) by decreasing the destructive effects of waste. Both earthworms and microorganisms involved in the process shows mutual relation and allows good waste biodegradation, thereby preserving the quality and nutritional value of the vermicompost [62]. Vermicompost based fertilizers are decomposed organic matter concentrations which help to improve crop productivity and soil fertility. Nutrients from waste materials are recycled and purified in this way, thus enhancing environmental health and its usage [63]. The vermicomposting method accompanies total benefit, i.e., its residual material can be utilized as a biofertilizer and can be practiced as a vermi-wash on plants for instance soybeans, cowpeas, maize, etc. thereby returning the nutrients from the soil back to the soil and so on [64]. While its length of time is very prominent and typically takes about 28–125 days and higher controllability requirements. Additionally, its operating system includes living organisms, and several conditions are required for their existence and formation of high-quality vermicompost. In general, a temperature range of 18–67 ℃, pH of 5.9–8.3, and a moisture content of 10.68% are required [65]. Thus, those obstacles limit the implementation of large-scale commercialization that needs to be overcome.

#### 2.2.3. Biofertilizers

Biofertilizers known as microbial inoculants are the carrier-based materials that contain beneficial microbes intended to enhance soil fertility and help the development of plants by increasing its diversity and biological activity [66]. It plays a vital role in decreasing inorganic fertilizer usage while increasing production capacity, in addition to preserving soil fertility. Bio-fertilizers are based on sources of renewable energy including apple orchard waste and are environmentally friendly in comparison to fertilizers from the industry. Fruit crops have typically obtained more attention in comparison to ornamental and vegetable crops [67]. A recent practice is the use of organic fertilizers to increase the production of fruit crops, it restricts chemical interference and eventually reduces the harmful effects on the wider climate [67]. Also, they serve as a relief to the poor farmers who cannot go with the expensive chemical fertilizers and the most useful organic matter added to the soil is high-quality compost and vermicompost.

Additionally, biochar is the carbonaceous solid product generated by the thermochemical transformation of organic substances, such as manure, apple leaves, and branches in an oxygen-depleted atmosphere that have properties appropriate for long-term carbon storage in the environment [68]. Biochar has a slow rate of decomposition that persists in the soil for more than 100 years when applied to the ground and has become one of the best choices for carbon sequestration in soil [69]. Agricultural use of biochar began in pre-Columbian times when slow vegetation burning was used to create nutrient-rich terra-preta soil. Biochar effectively improves the nitrogen utilization rate from 6.77% to 261.53% of the *Malus hupehensis*, and apple branch biochar has great potential to sequester carbon and is also favorable for soil fertility [70]. Furthermore, biochar increases the moisture-holding capacity and the soil nutrients by increasing the potential for cation exchange when applied in the soil. Global production of biochar is estimated in between 0.05 and 0.3 Gt C yr ^−1^ [71]. Lehmann et al. [72] estimated that carbon stored in the soil by using biochar could amount to 9.5 billion tones by 2100. Biochar production development for apple orchards is such a field that is therefore worth following.

### 2.3. Biochemicals recovery

**Figure 3.**
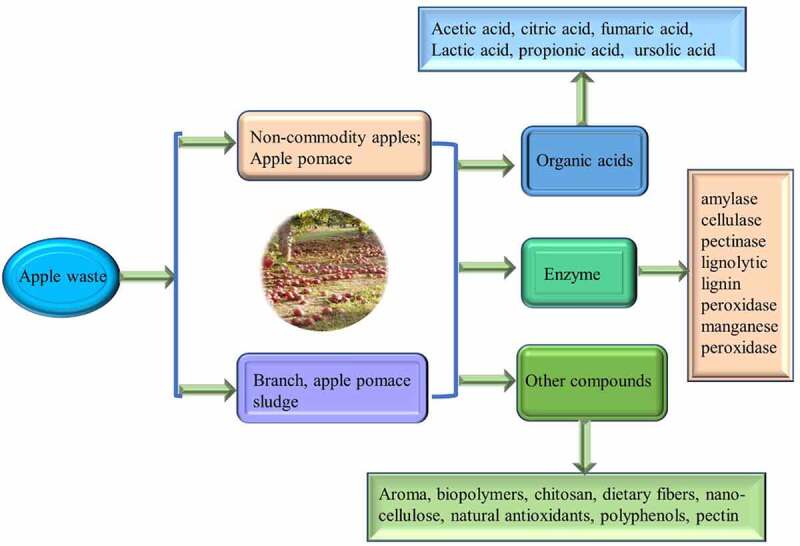
Biochemical recovery from apple orchard waste and its utilization in different forms

The biotechnological approach can convert the apple orchard waste into various value-added chemicals. Apple waste can be transformed into organic acids, aroma compounds, enzymes, edible dietary fibers, pectin, antioxidants, polyphenol, nano-cellulose, and bio-polymers [73]. Moreover, apple waste is reflected as a potential source for various environmental applications, especially for the removal of textile dye and heavy metal [74]. In this context, the potential of apple wastes summarized for the production of different bio-products in subsections using different biotechnological approach (Figure 2). Figure 3 shows the biochemical recovery from apple orchard waste and its utilization in different forms.

#### 2.3.1. Organic acid

**Figure 4.**
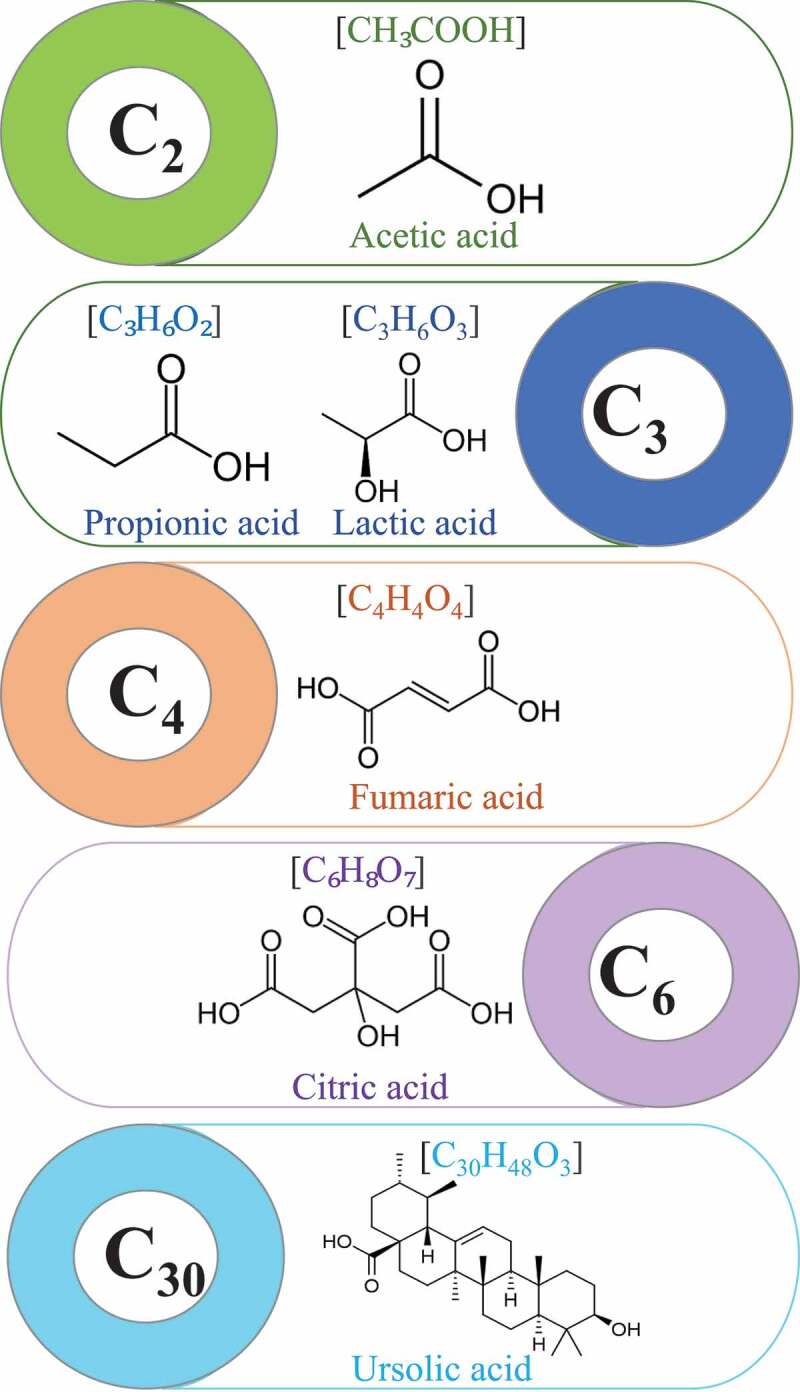
The chemical structure and formula of various organic acids

Currently, the organic acid is considered as platform chemicals due to several applications in cosmetics, detergent, food, pharmaceutical, polymer, and textile sector [75]. Globally, the organic acid market was assessed around $6.94 million by 2016 and it is projected to rise to $12.54 billion by 2026 [75,76]. Organic acids can be produced via chemical and biological routes. However, the production of organic acids via chemical routes offer higher productivity as well as yield but caused several environmental problems. While the biological routes offer several environmental benefits such as utilizing organic waste as well as emitting less pollutants. This demands a higher production cost due to substrate cost and lower production efficiency [77]. Therefore, the high production cost can be minimized using low-cost organic substrate as feedstock for the production of organic acid to achieving successful industrialization. Apple orchard waste can be considered as a potential feedstock for organic acid production due to the biodegradable properties of apple orchard waste and Figure 4 summarizes the potential of apple waste for the production of various organic acids.

Acetic acid (AA) is known as ethanoic acid. Its demand is increasing year by year globally due to high commercial importance [78]. Global demand for AA market is expected to rise with compound annual growth rates (CAGR) of 4.30% to 6.8% [79]. Almost, 75% of the AA is produced via chemical routes, while biological routes produce around 10% of the AA [80]. However, biological routes are considered as an expensive process but recognized as a clean and green process. In biological routes, microbial fermentation is carried out by a different group of microorganisms to produce AA, and leftovers after fermentation is used as animal feed or biofertilizer, *Acetobacteriaceae, Acetobacter, Gluconobacter*, and *Gluconoacetobacter* are commonly used for commercial production of AA [45]. Moreover, co-culture of different microbes is used to achieve higher productivities of AA [81]. Recently, Vashisht et al. [78] studied the potential of *Acetobacter pasteurianus* SKYAA25 for the conversion of apple waste into AA and highlighted the production of around 5.2 g of AA/10 g of dry biomass of apple pomace. Moreover, apple waste can be utilized for simulation production of organic acids and biofuels by acetone-butanol-ethanol (ABE) fermentation, which could decrease the AA production cost [82]. Besides, apple waste is considered as a suitable feedstock for citric acid (CA) production due to the lower pH (3–3.5) of apple waste by fungal strain *Aspergillus niger*, and that was known as the proficient producer of CA under acid conditions [83]. Dhillon et al. [83] reported that *A. niger* can produce around 3.42–3.65 g of AA/10 g dry biomass of apple waste. Nowadays, 99% of the CA produced by the fermentation process, while ~80% of the CA is produced via submerged fermentation globally, which due to safer and eco-friendly nature of the fermentation process [84]. The non-toxic, biodegradable, and biocompatible properties of natural CA allow the synthesis of biopolymers for biomedical applications [40]. Therefore, apple waste can be considered as a potential feedstock for CA production via the fermentation process to reduce the overall cost of production of CA biorefinery.

Additionally, fumaric acid (FA) is a chemical commodity that have different application in various sectors agricultural, chemical, leather, pharmaceutical, rubber, and textile sector. Additionally, due to non-toxic nature and unique flavor of FA, it has been commonly used in the food sector as an acidulant and beverage ingredient [85]. Furthermore, FA has been recognized as one of the top 10 platform chemical due to its various application, while the global production was 225.2 kt in 2012, with an estimated production of ~346 kt by 2020 with an expected compound annual growth rate (CAGR) of 5.9% from 2014 to 2020 [86,87]. Presently, FA is mostly produced via chemical methods, while biological methods are highly considered due to sustainability and eco-friendly nature [85]. During the biological production of FA various types of bacteria (*Bacillus macerans, Erwinia chrysanthemi, Thermoanaerobacter ethanolicus,* and *Zymomonas Mobilis*), yeasts (*Brettanomyces, Candida utilis, Pachysolen tannophilus* and *Scheffersomyces stipites*) and fungi (*Aspergillus glaucus, Caldariomycels fumago, Cirnella* spp., *Cunninghamella, Penicillum griseofulvum Rhizopus nigricans, R. arrhizus, R. oryzae,* and *R. formosa*) are involved [85]. Recently, Martin–Dominguez et al. [87] reviewed the biorefinery perspective of FA and summarized that *R. arrhizus* can produce to 121.0 g L^−1^ FA using glucose and nutrients as feed-stock, which bioprocess cost comparatively higher. Das et al. [88] accessed the potential of apple waste for FA production using *Rhizopus oryzae* 1526 and concluded that around 25.2 g L^−1^ FA can be produced in 72 h using 40 g/L of total solid concentration of apple waste during pH 6.0, 30°C, 200 rpm flask shaking speed. Therefore, apple waste can be considered as a cheap feed-stock for FA production via a fermentation process.

Lactic acid (LA) has extensive applications in the food, leather, pharmaceutical, and textile industries chemical feedstock. It can be produced via the chemical and biological process. During the biological synthesis process, various types of microorganisms and feed-stock (pure substrate and organic waste) is involved. However, the high cost of pure substrate force to search for alternative low-cost substrates. *Lactobacillus rhamnosus CECT-288* produces around 32.5 g/L of LA-LA after 6, while the mass balances showed that 4.6 g of LA can be produced from 10 kg of dry apple pomace by sequential hydrolysis and fermentation [89]. Moreover, propionic acid (PA) production from apple was studied by Piwowarek et al. and the result showed that 0.2 mg/L dose of biotin allowed to attain 7.66 g/L propionate [90]. Among several organic acids, ursolic acid (UA) has been considered for higher therapeutic properties such as anti-inflammatory, antitumor, antiprotozoal, and antibacterial [91]. Cargnin and Gnoatto [92] reviewed the potential of apple waste for UA production.

#### 2.3.2. Enzyme

Enzymes are known as bio-catalysts and has several potential applications in different industrial sectors. Due to the growing demand for enzymes, the global market for industrial enzymes is expected to reach around $6.3 billion in 2021 with a CAGR of 4.7% between 2016 and 2021 [93]. Hence, enzymes are comparatively expensive reagents, which increase the product cost of utilization. Therefore, the production cost of various enzymes needs to be reduced, which could be possible by using low-cost feedstock for enzyme production. In this regard, apple waste can be exploited for the production of various types of commercial enzymes via microbial bioprocess. Salim et al. reported that bacterial species *Bacillus* can produce α-amylase, protease, cellulase, and pectinase from different agricultural residues [94]. Kaur et al. [95] used apple waste as a substrate for amylase production during solid-state fermentation (SSF) using plant pathogenic fungus *Macrophomina phaseolina*. The maximum amylase activity of 3309.45 ± 29.22 IU/gds is observed at 35°C at 70% (v/w) initial moisture level after 120 h. Considering the importance of amylases in food and other industries, the huge amount of apple pomace and non-commercial apple as well as the convenience of obtaining apple can be used for the economical production of these enzymes to fulfill the needs of industries for the development of cost-effective processes. Moreover, apple waste is identified as a potential source for pectinase production, which various application in the industrial process for pectin degradation [96]. In the global enzyme sales, around 25% are contributed by pectinase [97]. Pectinases are classified into three types as i) pectin-esterase, ii) depolymerizing enzymes, which incorporate poly-methylgalacturonase and polygalacturonase, and iii) proto-pectinase, which solubilizes proto-pectin forming highly polymerized soluble pectin [98]. Pectinase enzymes has an important role in the industrialization era for extraction and clarification of fruit juice, application in alcoholic beverages and food industries, and several applications [97]. Table 2 summarizes the potential application of pectinase in various industrial sectors. Recently, Kuvvet et al. explored the potential of apple waste for low-cost enzyme (pectinases) production using the co-culture of *Bacillus subtilis* and *B. pumilus* [96]. The optimal conditions showed the maximum activity of pectinase was 11.25 IU mL^−1^ with 15% apple pomace (solid load), pH 9.0, and one-fourth of culture ratio (*B. subtilis*/and *B. pumilus*) at 30°C after 24 h.

**Table 2.** Industrial uses of pectinases at various stages of application [119].

Type of industry	Role	Applications
Animal feed	Reduces viscosity of feed	Rruminant feed
Brewing	Improve stability, chromaticity and luminosity	Beer clarification, chromatic
Coffee and tea fermentation	Removes mucilaginous coat and accelerates fermentation on process	Removal of coat and foam from coffee beans and tea
Food	Improve viscosity and stability	Clarification or gelling agent
Oil	Enzymatic extractions	Oil preparation
Pulp	Cell wall softening, bleaching	Depolymerization agent, maceration
Pharmaceutical	Controlled drug release	Drug delivery systems
Textile	Enhance fabric absorbency, decompose non-cellulosic material	Bio scouring, retting of plant bast fibres

Lignin-degrading enzyme have a significant role in the degradation of lignin and persistent organic pollutants (POPs) [99]. Mostly, the lignin-degrading enzymes can be produced by various microorganisms including fungi, bacteria, and actinomycetes, among which fungi play a more prominent role during the lignin degradation [100,101]. Moreover, lignocellulose waste of apple branch and leaves contain an ample amount of lignin, which is an amorphous aromatic polymer with a high molecular weight. Due to the complex structure, it is hard to degrade lignin, which demands an efficient degradation substance for lignin degradation. Amongst various lignin degradation methods biological methods highly considered due to low energy requirements, mild operating conditions, and considered as eco-friendly as compared with physical and chemical methods [102,103]. Several enzymes such as aryl alcohol oxidase, aryl alcohol dehydrogenase, cellobiose dehydrogenase, laccase (Lac), lignin peroxidase (LiP), and manganese peroxidase (MnP) are recognized for lignin degradation. Among them, Lac, LiP, and MnP are highly considered for efficient lignin degradation [101]. Gassara et al. examined the potential of apple pomace for the production of lignolytic enzyme *Phanerochaete chrysosporium BKM-F-1767* under solid-state fermentation conditions [104]. During the ligninolytic enzyme production various types of enzyme work as inducers, such as veratryl alcohol, Tween-80, and CuSO_4_ at concentrations of 2 mM, 0.5% (v/w), and 3 mmole/kg, respectively, are added during the solid-state fermentation. The result showed that the addition of veratryl alcohol and Tween-80 resulted in maximum manganese peroxidase (MnP) activity of 17.36, 540.2, 631.25, and 507.5 26.87 U/gds (units/gram dry substrate). While maximum lignin peroxidase (LiP) activity of 141.38 is attained. Extension of earlier reported study is carried out by Gassara et al. to achieve enhanced production of LiP, MnP, and Lac production using apple waste by *P. chrysosporium* [105]. Various experimental effects such as the role of moisture, copper sulfate, and veratryl alcohol (VA) concentrations on ligninolytic enzyme production is excessed. Moreover, experimental outcomes showed enhanced MnP and LiP productivity in the presence of moisture and VA, while the availability of moisture and copper sulfate enhanced on Lac productivity. Therefore, apple waste is a potential source for various enzyme production.

#### 2.3.3. Other compounds

Apple waste can be explored in biorefinery processes for obtaining aroma compounds, biopolymers, chitosan, dietary fibers, microbial oils, nano-cellulose, natural antioxidants, polyphenols, and pectin’s [106,107]. Among them natural antioxidants and polyphenols are highly considered as antioxidant, antimicrobial, anti-inflammatory, anticarcinogenic, and cardiovascular protective properties for food, pharmaceutical, and cosmetic applications [108,109]. Aroma compounds have an imperative role in the food sector, which comprises 25% of the global demand as food additives [110]. Mostly the aroma compounds are synthesized via chemical methods and biological methods (Extraction from natural matrixes and biosynthesis or bioconversion via different microbes). Chemically synthesized aroma compounds are not considered nowadays due to health awareness, natural aroma compounds highly considered due to several benefits [73]. Therefore, the synthesis of natural aroma compounds via biological routes is highly recommended. Rodríguez et al. utilized the apple pomace for the production of volatile aroma compounds via fermentation by *Hanseniaspora uvarum, Hanseniaspora valbyensis,* and *Saccharomyces cerevisiae*, the result showed that apple pomace can be produced around 132 volatile aroma compounds, which was strongly strain dependent [110]. Ricci et al. reported that apple pomace could be utilized by lactic acid bacteria for production aromatize for alcoholic beverages [111]. Moreover, apple waste can be exploited for the production of biopolymers via a microbial fermentation process. Recently, Rebocho et al. explored the potential of apple waste for the production of poly(3-hydroxybutyrate), P(3HB), and medium-chain length polyhydroxyalkanoates (PHA), mcl-PHA by using the co-culture of *Cupriavidus necator* DSM 428 and *Pseudomonas citronellolis* NRRL B-2504 [112]. The biopolymers accumulated by both strains and this study demonstrates the feasibility of apple waste for the production of a natural blend of P(3HB)/mcl-PHA. Also, chitosan (CS) is recognized as a bio-polymer due to its non-toxic, biodegradable, biocompatible, and intrinsic antimicrobial properties, which is as a de-acetylated derivative of chitin [113,114]. However, there are limited literature that have been explored the potential of apple waste for CS production. Vendruscolo et al. utilized the apple pomace for fungal chitosan production in an external-loop airlift bioreactor by Gongronella butleri, the effect of the nitrogen sources NaNO_3_ and (NH_4_)_2_SO_4_ and specific aeration rate of 0.1, 0.3, and 0.6 vvm are investigated, the result showed that ammonium sulfate and specific aeration rate of 0.6 vvm had a significant effect on chitosan production [115]. Moreover, the apple waste has the potential for the production of other bioactive compounds such as dietary fibers, microbial oils, nano-cellulose, natural antioxidants, polyphenols, and pectin’s, which compound have several applications in different properties as shown in Table 3.

**Table 3.** Major bioactive compounds from apple pomace [120]

Ingredient	Major Bioactive Compounds	Bioactivity and Therapeutic Potential
Carbohydrates	Pectin and oligosaccharides	Soluble viscous fermentable fiber/dietary fiber, potential prebiotic properties
Phenolic acids	P-coumaric acid sinapic, p-coumaroyl-quinic, caffeic, ferulic and chlorogenic	Soluble viscous fermentable fiber/dietary fiber, potential prebiotic properties
Flavonoids	Isorhamnetin, procyanidinB2, kaempferol, rhamnetin, glycoconjugates, guercetin, epicatechin	Antioxidant, antiinflammatory, anticancer and cardio-protective
Anthocyanins	Cyanidin-3-O-galactoside	Antioxidant, antiinflammatory, anticancer and cardio-protective
Dihydrochalcones	Phlorizin, phloretein	Antidiabetic, promoting bone-forming, blastogenesis
Triterpenoids	Ursolic and oleanolic acid	Antimicrobial and antiinflammatory

## 3. Current challenges and future direction of recycling apple orchard waste

Apple orchard waste degradation is producing a large flow of nutrients back to the soil. The nutrient flux associated with the decomposition of litter has gained a lot of attention in both natural and semi-natural ecosystems. This represents the main nutrient source needed to sustain soil fertility, while the ecosystems in orchards have been poorly considered so far. Currently, disposal of apple-dropping or woody biomass is done by natural degradation or mulching in the field of by direct on-site combustion. In both circumstances, drawbacks and costs are significantly incurred by the farmer: law prohibits open combustion while the risk of fungal diseases may be increased due to leaving residues on the ground. Due to the uncomplimentary ratio of C:N ratio of woody residues, the reduction of nitrogen availability in the crop is another problem, which contains an upsurge in input of fertilization. While production of fruits in a sustainable manner whether organic or integrated now shows a growing trend to restrict the amount of fertilizer to be used by farmers. As the value rises for inorganic fertilizers, there is a terrible requirement to utilize the generated waste as a source of nutrient recovery. Organic and inorganic fertilizers may serve as an effective option for meeting increasing nutrient demands and addressing the problems related to climate change and food security. Such as degradable apple and cellulose-rich orchard woody waste-composting can be utilized as a source of essential nutrients production from agricultural crop and convert into organic matter to achieve nutrient recovery. However, the current penetration rate still needs to be improved, and the lack of commercialization was limited by the high cost of logistics, recycling and classification processing, and insufficient awareness. Therefore, classified management and collection of waste are extremely important, although China has already carried out the classification and recycling of some domestic waste, but the agricultural sector is still need to strengthen implementation and extension.

As reported that heating value attained 5 MJ/kg and 20 MJ/kg for fresh and dry wood residues that equivalent to 40% of the calorific value of coal combustion. Biomass can be used as a biofuel by collecting and processing into an appropriate shape, while this feedstock is usually not suitable for Thermoelectric combination Combined Heat and Power Generation (CHP) and large power plants due to its high logistic costs, which are usually spread over low-density landscapes, and these power plants typically depend on imported biomass [116]. However, AOW can be effectively installed in smaller inhabited heating systems in which domestic biomass is primarily used, reducing reliance on the source of imported fossil energy, resource chains with a lower social and environmental impact. According to local conditions, farmers in geographically inconvenient areas should be treated nearby, and conditions that allow for reasonable treatment of waste in the area should be resource recycling by disposal and management. Relevant departments should certain extent popularize relevant infrastructure technology according to regional characteristics, advocate the principle of near handling and provide service and support.

In terms of bio-chemicals and biofuels recovery, such as for the production of biobutanol and bioethanol, apple waste can be used as a potential substratum because of its high accessibility of starch, cellulose content, and non-competitiveness with food security [117]. Although the potential of apple-plated biomass for energy purposes is immeasurable, nevertheless, it requires a certain amount of energy supply and manpower production as well as expenditure in essential machinery. In common terms, the specific biofuel composition differs with the composition and source of decomposable biomass, the fuel shall have a similar and satisfactory distribution of a low ash content and the particle size. Therefore, the absolute suitable quality of the biomass classification is another challenge needed to breakthrough.

Manufacturers are now endeavoring to build machines that help to manage wood content and technology of reaction equipments. Flexible bioreactors of continuous-stirred tank reactors could be used for uniform biomass mixing by the way of a mechanical agitator or biogas recycling contributing to the same hydraulics retention time and therefore to an increased average biogas yield. Molinuevo-Salces et al. [118] used a continuous stirred-tank reactor to study the effects of the addition of vegetable waste (50% dw/dw) in swine manure anaerobic digestion that resulted in increased methane yield. Therefore, integrate feasible technologies to maximize the diversity of benefits while being more economical and practical, realize the clean production of AOW, resource utilization in multiple ways, and achieve sustainable development.

## 4. Conclusion

Apple orchard waste comprises of various available carbohydrate-rich biomass (branch, leaves, and non-commercial apple) and hence it is proposed as a green or alternative bioresource in the form of diverse value-added products. Finally, to achieve the energy recovery aspect of producing green and clean alternative fuels, chemical, and nutrient recovery-based on safe and harmless biochemical and biofertilizer production is realized. While the expensive cost of investment needs to rely on continuous improvement of technology, it is still facing various uneven technical and social challenges. The incentives of related policies, the improvement of commerce, and national awareness are necessary for the popularization and commercialization of products.

## Abbreviation

Acetic acid - AA

Acetone-butanol-ethanol - ABE

Apple orchard waste - AOW

Carbon/nitrogen - C/N

Carbon dioxide - CO_2_

Chitosan - CS

Citric acid - CA

Combined heat and power generation - CHP

Compound annual growth rates - CAGR

Continuous stirred tank reactors - CSTR

Fumaric acid - FA

Higher heating value - HHV

Hydraulic retention time - HRT

Hydrogen - H_2_

Laccase - Lac

Lactic acid - LA

Lignin peroxidase - LiP

Manganese peroxidase - MnP

Nitrous oxide - N_2_O

Persistent organic pollutants - POPs

Polyhydroxyalkanoates - PHA

Propionic acid - PA

Pruning-to-energy - PtE

Renewable energy sources - RES

Ursolic acid - UA

Veratryl alcohol – VA

## Highlights

1. Apple orchard waste (AOW) biorefinery served as sustainable waste recycling strategies.
2. AOW recycling have distinct biorefinery approaches and bioproduct according to the component.
3. AOW recycling could be contributed to biofuels, nutrient, and biochemical recovery.
4. Biorefinery is facing a big challenge in technical, financial, and social awareness.
5. AOW recycling with circular bioeconomy requires coordinated policy intervention and socioeconomic aspect.

## References

[1] Cruz MG, Bastos R, Pinto M, et al.. Waste mitigation: from an effluent of apple juice concentrate industry to a valuable ingredient for food and feed applications. J Clean Prod. 2018;193:652–660.

[2] Barnwal BK, Sharma MP. Prospects of biodiesel production from vegetable oils in India. Renew. Sustain. Energy. Rev. 2005;9(4):363–378.

[3] Nati C, Boschiero M, Picchi G, et al.. Energy performance of a new biomass harvester for recovery of orchard wood wastes as alternative to mulching. Renew. Energ. 2017;124:121–128.

[4] Wang X, Kristo E, Lapointe G. The effect of apple pomace on the texture, rheology and microstructure of set type yogurt. Food. Hydrocolloid. 2019;91:83–91.

[5] Al-Hamamre Z, Saidan M, Hararah M, et al.. Wastes and biomass materials as sustainable-renewable energy resources for Jordan. Renew. Sustain. Energy. Rev. 2017;67:295–314.

[6] Avcioglu AO, Dayioglu MA, Turker U. Assessment of the energy potential of agricultural biomass residues in turkey. Renew. Energ. 2019;138:610–619.

[7] Alavijeh MK, Yaghmaei S. Biochemical production of bioenergy from agricultural crops and residue in iran. Waste. Manage. 2016;52:375–394. 10.1016/j.wasman.2016.03.025 27012716

[8] Conti J, Holtberg P，Diefenderfer J, et al. International energy outlook 2016, in: with Projections to 2040, U.S. Energy Information Administration Office of Energy Analysis U.S, Department of Energy, Washington DC. 2016;290: 20585.

[9] Kumar A, Kumar K, Kaushik N, et al.. Renewable energy in India: current status and future potentials. Renew. Sustain. Energy. Rev. 2010;14(8):2434–2442.

[10] Brand M, Jacinto R. Apple pruning residues: potential for burning in boiler systems and pellet production. Renew. Energ. 2020;152:458–466.

[11] Zlatanovic S, Ostoji S, Mici DM, et al.. Thermal behaviour and degradation kinetics of apple pomace flours. Thermochimica. Acta. 2019;673:17–25.

[12] Rupasinghe HPV, Kean C, Nichols D, et al.. Orchard waste as a valuable bio-resource: a chemical composition analysis. Acta Horticult. 2007;737(737):17–23.

[13] Awasthi MK, Sarsaiya S, Wainaina S, et al.. A critical review of organic manure bio-refinery models toward sustainable circular bio-economy: technological challenges, advancements, innovations, and future perspectives. Renew. Sustain. Energy. Rev. 2019;111:115–131.

[14] Taherzadeh MJ. Bioengineering to tackle environmental challenges, climate changes and resource recovery. Bioengineered. 2019;10(1):698–699. 31847689 10.1080/21655979.2019.1705065 PMC8530266

[15] García R, Pizarro C, Lavín AG, et al.. Spanish biofuels heating value estimation. Part II: proximate analysis data. Fuel. 2014;117:1139–1147.

[16] EUROSTAT Sustainable Development in the European Union :Monitoring Report of the EU Sustainable Development Strategy;Publications Office of the European Union:Luxembourg;2011.

[17] García-Galindo D, Cay Villa-Ceballos F, Vila-Villarroel L, et al. Seeking for ratios and correlations from field data for improving biomass assessments for agricultural pruning in Europe. Method and Results. In Proceedings of the 24th European Biomass Conference and Exhibition, Amsterdam, The Netherlands. 2016; 6–9.

[18] Uzodinma EO, Ofoefule AU, Enwere NJ. Optimization of biogas fuel production from maize (Zea mays) bract waste: comparative study of biogas production from blending maize bract with biogenic wastes. Am. J. Food. Nutr. 2011;1(1):1–6.

[19] Bouallagui H, Touhami Y, Cheikh RB, et al.. Bioreactor performance in anaerobic digestion of fruit and vegetable wastes. Process. Biochem. 2005;40(3–4):989–995.

[20] Guo S, Lu C, Wang K, et al.. Enhancement of pH values stability and photofermentation bio-hydrogen production by phosphate buffer. Bioengineered. 2020;11(1):291–300. 32129696 10.1080/21655979.2020.1736239 PMC7161566

[21] Wainaina S, Lukitawesa AMK, Taherzadeh MJ. Bioengineering of anaerobic digestion for volatile fatty acids, hydrogen or methane production: A critical review. Bioengineered. 2019;10(1):437–458. 31570035 10.1080/21655979.2019.1673937 PMC6802927

[22] Li K, Liu R, Cui S, et al.. Anaerobic co-digestion of animal manures with corn stover or apple pulp for enhanced biogas production. Renew. Energ. 2018;118:335–342.

[23] Jansson AT, Patinvoh RJ, Taherzadeh MJ, et al.. Effect of organic compounds on dry anaerobic digestion of food and paper industry wastes. Bioengineered. 2019;11(1):502–509. 10.1080/21655979.2020.1752594 PMC7185885 32303143

[24] Kafle G, Kim S. Anaerobic treatment of apple waste with swine manure for biogas production: batch and continuous operation. Appl Energy. 2013;103:61–72.

[25] Xiao W, Li H, Xia W, et al.. Co-expression of cellulase and xylanase genes in Sacchromyces cerevisiae toward enhanced bioethanol production from corn stover. Bioengineered. 2019;10(1):513–521. 31661645 10.1080/21655979.2019.1682213 PMC6844370

[26] Karmakar A, Karmakar S, Mukherjee S. Properties of various plants and animals’ feed-stocks for biodiesel production. Bioresource. Technol. 2010;101(19):7201–7210. 10.1016/j.biortech.2010.04.079 20493683

[27] Diyauddeen BH, Aziz AA, Daud W. Chakrabarti M. Performance evaluation of biodiesel from used domestic waste oils: a review. Process. Saf. Environ. 2012;90(3):164–179.

[28] Atadashi IM, Aroua MK, Aziz AA, et al.. Production of biodiesel using high free fatty acid feedstocks. Renew. Sustain. Energy. Rev. 2012;16(5):3275–3285.

[29] Li S, Wang Y, Dong S, et al.. Biodiesel production from Eruca Sativa Gars vegetable oil and motor, emissions properties. Renew. Energ. 2009;34(7):1871–1876.

[30] Su W, Ma H, Gao M, et al. Research on biodiesel and ethanol production from food waste. In 2010 4th International Conference on Bioinformatics and Biomedical Engineering. china. 2010.

[31] Pizarro AVL, Park EY. Lipase-catalyzed production of biodiesel fuel from vegetable oils contained in waste activated bleaching earth. Process. Biochem. 2003;38(7):1077–1082.

[32] Keera ST, El Sabagh SM, Taman AR. Transesterification of vegetable oil to biodiesel fuel using alkaline catalyst. Fuel. 2011;90(1):42–47.

[33] Shu Q, Gao J, Nawaz Z, et al.. Synthesis of biodiesel from waste vegetable oil with large amounts of free fatty acids using a carbon-based solid acid catalyst. Appl Energy. 2010;87(8):2589–2596.

[34] Aguilar C, Ruiz H, Rios A, et al.. Emerging strategies for the development of food industries. Bioengineered. 2019;10(1):522–537. 31633446 10.1080/21655979.2019.1682109 PMC6844418

[35] Li P, He C, Li G, et al.. Biological pretreatment of corn straw for enhancing degradation efficiency and biogas production. Bioengineered. 2020;11(1):251–260. 32125259 10.1080/21655979.2020.1733733 PMC7161559

[36] Lee S, Posarac D, Ellis N. An experimental investigation of biodiesel synthesis from waste canola oil using supercritical methanol. Fuel. 2012;91(1):229–237.

[37] Sandhu DK, Joshi VK. Solid state fermentation of apple pomace for con- comitant production of ethanol and animal feed. J. Sci. Ind. Res. 1997;56:86–90.

[38] Ngadi MO, Correia LR. Solid state ethanol fermentation of apple pomace as affected by moisture and bioreactor mixing speed. J. Food. Sci. 1992;57(3):667–670.

[39] Dhillon GS, Kaur S, Brar SK. Perspective of apple processing wastes as low-cost substrates for bioproduction of high value products: A review. Renew. Sustain. Energy. Rev. 2013;27:789–805.

[40] Zhang S, Wang Y, Liu S. Process optimization for the anaerobic digestion of poplar (Populus L.) leaves. Leaves. Bioengineered. 2020;11(1):439–448. 32189559 10.1080/21655979.2020.1739823 PMC7161560

[41] Lütke-Eversloh T, Bahl H. Metabolic engineering of Clostridium acetobutylicum: recent advances to improve butanol production. Curr Opin Biotech. 2011;22(5):634–647. 21377350 10.1016/j.copbio.2011.01.011

[42] Voget CE, Mignone CF, Ertola RJ. Butanol production from apple pomace. Biotechnol Lett. 1985;7(1):43–46.

[43] Patakova P, Lipovský J, Cížková H, et al.. Exploitation of food feedstock and waste for production of biobutanol. Czech. J. Food. Sci. 2009;27(No. 4):276–283.

[44] Gomes RJ, Borges M, Rosa M, et al.. Acetic acid bacteria in the food industry: systematics, characteristics and applications. Food. Technol. Biotech. 2018;56(2):139–151. 10.17113/ftb.56.02.18.5593 PMC6117990 30228790

[45] Shi T, Peng H, Zeng S, et al.. Microbial production of plant hormones: opportunities and challenges. Bioengineered. 2017;8(2):124–128. 27459344 10.1080/21655979.2016.1212138 PMC5398602

[46] Show KY, Lee DJ, Chang JS. Bioreactor and process design for bio-hydrogen production. Bioresource. Technol. 2011;102(18):8524–8533. 10.1016/j.biortech.2011.04.055 21624834

[47] Kharkwal G. Qualitative analysis of tree species in evergreen forests of Kumaun Himalaya, Uttarakhand, India. African. J. Plant. Sci. 2009;3:049–052.

[48] Mohanakrishna G, Goud RK, Mohan SV, et al.. Enhancing biohydrogen production through sewage supplementation of composite vegetable-based market waste. Int J Hydrogen Energy. 2010;35(2):533–541.

[49] Yao B, Xiao T, Jie X, et al.. H2–rich gas production from leaves. Catal Today. 2018;317:43–49.

[50] Tenca A, Schievano A, Perazzolo F, et al.. Biohydrogen from thermophilic co-fermentation of swine manure with fruit and vegetable waste: maximizing stable production without pH control. Bioresource. Technol. 2011;102(18):8582–8588. 10.1016/j.biortech.2011.03.102 21530242

[51] Lukitawesa PR, Millati R, Horváth S, et al.. Factors influencing volatile fatty acids production from food wastes via anaerobic digestion. Bioengineered. 2019;11(1):39–52. 10.1080/21655979.2019.1703544 PMC7571609 31880192

[52] Lu C, Zhang Z, Ge X, et al.. Bio-hydrogen production from apple waste by photosynthetic bacteria HAU-M1. Int. J. Hydrogen. Energ. 2016;41(31):13399–13407.

[53] Tagliavini M, Tonon G, Scandellari F, et al.. Nutrient recycling during the decomposition of apple leaves (Malus domestica) and mowed grasses in an orchard. Agr. Ecosyst. Environ. 2007;118(1–4):191–200.

[54] Tartachnyk II, Blanke MM. Effect of delayed fruit harvest on photosynthesis, transpiration and nutrient remobilization of apple leaves. New. Phytol. 2004;164(3):441–450.

[55] Kopcic N, Domanovac MV, Kucic D, et al.. Evaluation of laboratory-scale in-vessel co-composting of tobacco and apple waste. Waste Manage. 2014;34(2):323–328. 10.1016/j.wasman.2013.11.001 24290970

[56] Mia S, Uddin ME, Kader MA, et al.. Pyrolysis and co-composting of municipal organic waste in Bangladesh: A quantitative estimate of recyclable nutrients, greenhouse gas emissions, and economic benefits. Waste. Manage. 2018;75:503–513. 10.1016/j.wasman.2018.01.038 29439929

[57] Venkatesh RM, Eevera T. Mass reduction and recovery of nutrients through vermicomposting of fly ash. Appl. Ecol. Env. Res. 2008;6(1):77–84.

[58] Sinha RK, Agarwal S, Chauhan K, et al.. The wonders of earthworms & its vermicompost in farm production: charles Darwin’s ‘friends of farmers’, with potential to replace destructive chemical fertilizers. Agr. Sci. 2010;1:76.

[59] Hanc A, Chadimova Z. Nutrient recovery from apple pomace waste by vermicomposting technology. Bioresource. Technol. 2014;168:240–244. 10.1016/j.biortech.2014.02.031 24582426

[60] García-Sánchez M, Tausnerova H, Hanc A, et al.. Stabilization of different starting materials through vermicomposting in a continuous-feeding system: changes in chemical and biological parameters. Waste. Manage. 2017;62:33–42. 10.1016/j.wasman.2017.02.008 28215973

[61] Singh RP, Embrandiri A, Ibrahim MH, et al.. Management of biomass residues generated from palm oil mill: vermicomposting a sustainable option. Resour. Conserv. Recy. 2011;55(4):423–434.

[62] Bhat SA, Singh S, Singh J, et al.. Bioremediation and detoxification of industrial wastes by earthworms: vermicompost as powerful crop nutrient in sustainable agriculture. Bioresource. Technol. 2018;252:172–179. 10.1016/j.biortech.2018.01.003 29321101

[63] Macdonald C, Singh B. Harnessing plant-microbe interactions for enhancing farm productivity. Bioengineered. 2014;5(1):5–9. 23799872 10.4161/bioe.25320 PMC4008467

[64] Manyuchi MM, Phiri A. Vermicomposting in solid waste management: a review. Int. J. Sci. Engineer. Technol. 2013;2:1234–1242.

[65] Ahad S, Mir M, Ashraf S, et al.. Nutrient Management in High Density Apple Orchards–A Review. Curr. J. Appl. Sci. Technol. 2018;29(1):1–16.

[66] Kumar A, Singh E, Khapre A, et al.. Sorption of volatile organic compounds on non-activated biochar. Bioresource. Technol. 2020;297:122469. 10.1016/j.biortech.2019.122469 31787517

[67] DeLuca TH, Gundale MJ, MacKenzie MD, et al.. Biochar effects on soil nutrient transformations. Biochar for Environmental Management: Science, Technology and Implementation. 2015;2:421–454.

[68] Li S, Liang C, Shangguan Z. Effects of apple branch biochar on soil C mineralization and nutrient cycling under two levels of N. Sci Total Environ. 2017;607-608:109. 28688253 10.1016/j.scitotenv.2017.06.275

[69] Navia R, Crowley DE. Closing the loop on organic waste management: biochar for agricultural land application and climate change mitigation. Waste Management & Research the Journal of the International Solid Wastes & Public Cleansing Association ISWA. 2010;28(6):479–480. 10.1177/0734242X10370928 20507863

[70] Atkinson CJ, Fitzgerald JD. Hipps NA. Potential mechanisms for achieving agricultural benefits from biochar application to temperate soils: a review. Plant Soil. 2010;337(1–2):1–18.

[71] Lehmann J, Gaunt J, Rondon M. Bio-char sequestration in terrestrial ecosystems–a review. Mitig. Adapt. Strat. Gl. 2006;11(2):403–427.

[72] Dhillon GS, Brar SK, Kaur S, et al.. Screening of agro-industrial wastes for citric acid bioproduction by Aspergillus niger NRRL 2001 through solid state fermentation. J. Sci. Food. Agric. 2013;93(7):1560–1567. 23108761 10.1002/jsfa.5920

[73] Chang Y, Lai JY, Lee DJ. Thermodynamic parameters for adsorption equilibrium of heavy metals and dyes from wastewaters: research updated. Bioresour Technol. 2016;222:513–516. 27720331 10.1016/j.biortech.2016.09.125

[74] Lu J, Lv Y, Qian X. et al.. Current advances in organic acid production from organic wastes by using microbial co-cultivation systems. Biofuel. Bioprod. Bio. 2020;14(2):481–492. DOI:10.1002/bbb.2075.

[75] Kim HM, Park JH, Choi IS, et al. Effective approach to organic acid production from agricultural kimchi cabbage waste and its potential application. PLoS One. 2018;13:1–14. 10.1371/journal.pone.0207801 PMC6245790 30458042

[76] Becker J, Lange A, Fabarius J, et al.. Top value platform chemicals: bio-based production of organic acids. Curr Opin Biotech. 2015;36:168–175. 26360870 10.1016/j.copbio.2015.08.022

[77] Vashisht A, Thakur K, Kauldhar BS, et al.. Waste valorization: identification of an ethanol tolerant bacterium Acetobacter pasteurianus SKYAA25 for acetic acid production from apple pomace. Sci Total Environ. 2019;690:956–964. 31302559 10.1016/j.scitotenv.2019.07.070

[78] Pal P, Nayak J. Acetic acid production and purification: critical review towards process intensification. Sep. Purif. Rev. 2017;46(1):44–61.

[79] De Roos J, De Vuyst L. Acetic acid bacteria in fermented foods and beverages. Curr Opin Biotech. 2018;49:115–119. 28863341 10.1016/j.copbio.2017.08.007

[80] Evcan E, Tari C. Production of bioethanol from apple pomace by using co-cultures: conversion of agro-industrial waste to value added product. Energy. 2015;88:775–782.

[81] Jin Q, Qureshi N, Wang H, et al.. Acetone-butanol-ethanol (ABE) fermentation of soluble and hydrolyzed sugars in apple pomace by Clostridium beijerinckii P260. Fuel. 2019;244:536–544.

[82] Dhillon GS, Brar SK, Verma M, et al.. Enhanced solid-state citric acid bio-production using apple pomace waste through surface response methodology. J Appl Microbiol. 2011;110(4):1045–1055. 21294819 10.1111/j.1365-2672.2011.04962.x

[83] Sun X, Lu H, Wang J. Recovery of citric acid from fermented liquid by bipolar membrane electrodialysis. J Clean Prod. 2017;143:250–256.

[84] Guo F, Wu M, Dai Z, et al. Current advances on biological production of fumaric acid. Biochem Eng J. 2020;153:107397.

[85] Papadaki A, Androutsopoulos N, Patsalou M, et al. Biotechnological Production of Fumaric Acid: the Effect of Morphology of Rhizopus arrhizus NRRL 2582. Fermentation. 2017;3(3):33.

[86] Martin-Dominguez V, Estevez J, Ojembarrena F, et al.. Fumaric Acid Production: A Biorefinery Perspective. Fermentation. 2018;4(2):33.

[87] Das RK, Brar SK, Verma M. A fermentative approach towards optimizing directed biosynthesis of fumaric acid by Rhizopus oryzae 1526 utilizing apple industry waste biomass. Fungal Biol. 2015;119(12):1279–1290. 26615750 10.1016/j.funbio.2015.10.001

[88] Gullón B, Yáñez R, Alonso JL, et al.. L-Lactic acid production from apple pomace by sequential hydrolysis and fermentation. Bioresour Technol. 2008;99(2):308–319. 17321133 10.1016/j.biortech.2006.12.018

[89] Piwowarek K, Lipinska E, Hac-Szymanczuk E, et al.. Optimization of propionic acid production in apple pomace extract with Propionibacterium freudenreichii. Prep Biochem Biotechnol. 2019;49(10):974–986. 31403887 10.1080/10826068.2019.1650376

[90] Zang L, Wu B, Lin Y, et al.. Research progress of ursolic acid’s anti-tumor actions. Chin J Integr Med. 2014;20(1):72–79. 24374755 10.1007/s11655-013-1541-4

[91] Cargnin ST, Gnoatto SB. Ursolic acid from apple pomace and traditional plants: A valuable triterpenoid with functional properties. Food Chemistry. 2017;220:477–489. 27855928 10.1016/j.foodchem.2016.10.029

[92] Ravindran R, Hassan SS, Williams GA, et al.. Review on Bioconversion of Agro-Industrial Wastes to Industrially Important Enzymes. Bioengineering. 2018;5:93. 10.3390/bioengineering5040093 PMC6316327 30373279

[93] Salim AA, Grbavcic S, Šekuljica N, et al.. Production of enzymes by a newly isolated Bacillus sp. TMF-1 in solid state fermentation on agricultural by-products: the evaluation of substrate pretreatment methods. Bioresour. Technol. 2017;228:193–200. 10.1016/j.biortech.2016.12.081 28063362

[94] Kaur S, Dhillon GS, Brar SK, et al.. Carbohydrate degrading enzyme production by plant pathogenic mycelia and microsclerotia isolates of Macrophomina phaseolina through koji fermentation. Ind Crops Prod. 2012;36(1):140–148.

[95] Kuvvet C, Uzuner S, Cekmecelioglu D. Improvement of Pectinase Production by Co-culture of Bacillus spp. Using Apple Pomace as a Carbon Source. Waste. Biomass. Valori. 2019;10(5):1241–1249.

[96] Sharma R, Oberoi HS, Dhillon GS. Chapter 2 - Fruit and Vegetable Processing Waste: renewable Feed Stocks for Enzyme Production,” in Agro-Industrial Wastes as Feedstock for Enzyme Production. Dhillon GS, Kaur S, eds. (San Diego: Academic Press); 2016. p. 23–59.

[97] Favela-Torres E, Volke-Sepúlveda T, Viniegra-González G. Production of Hydrolytic Depolymerising Pectinases. Food. Technol. Biotech. 2006;44(2):221–227.

[98] Huang S, Huang D, Wu Q, et al.. Effect of environmental C/N ratio on activities of lignin-degrading enzymes produced by Phanerochaete chrysosporium. Pedosphere. 2020;30:285–292.

[99] Asgher M, Wahab A, Bilal M, et al.. Lignocellulose degradation and production of lignin modifying enzymes by Schizophyllum commune IBL-06 in solid-state fermentation. Biocatal. Agric. Biotechnol. 2016;6:195–201.

[100] Zhang S, Xiao J, Wang G, et al.. Enzymatic hydrolysis of lignin by ligninolytic enzymes and analysis of the hydrolyzed lignin products. Bioresour Technol. 2020;304:122975. 32086036 10.1016/j.biortech.2020.122975

[101] Wang J, Cui Z, Li Y, et al.. Techno-economic analysis and environmental impact assessment of citric acid production through different recovery methods. J Clean Prod. 2020;249:119315.

[102] Bilal M, Iqbal H, Hu H, et al.. Metabolic engineering and enzyme-mediated processing: A biotechnological venture towards biofuel production – A review. Renew. Sustain. Energy. Rev. 2018;82:436–447.

[103] Gassara F, Brar SK, Tyagi RD, et al.. Screening of agro-industrial wastes to produce ligninolytic enzymes by Phanerochaete chrysosporium. Biochem Eng J. 2010;49(3):388–394.

[104] Gassara F, Brar SK, Tyagi RD, et al.. Parameter optimization for production of ligninolytic enzymes using agro-industrial wastes by response surface method. Biotechnol. Bioproc. E. 2011;16(2):343–351.

[105] Vodnar DC, Calinoiu LF, Dulf FV, et al.. Identification of the bioactive compounds and antioxidant, antimutagenic and antimicrobial activities of thermally processed agro-industrial waste. Food Chem. 2017;231:131–140. 28449989 10.1016/j.foodchem.2017.03.131

[106] Guardia L, Suárez L, Querejeta N, et al.. Apple Waste: A sustainable source of carbon materials and valuable compounds. ACS. Sustain. Chem. Eng. 2019;7(20):17335–17343.

[107] Perussello CA, Zhang Z, Marzocchella A, et al.. Valorization of apple pomace by extraction of valuable compounds. Compr. Rev. Food. Sci. Food. Saf. 2017;16(5):776–796. 33371603 10.1111/1541-4337.12290

[108] Waldbauer K, McKinnon R, Kopp B. Apple pomace as potential source of natural active compounds. Planta Medica. 2017;83(12/13):994–1010. 28701021 10.1055/s-0043-111898

[109] Rodríguez Madrera R, Pando Bedriñana R, Suárez Valles B. Production and characterization of aroma compounds from apple pomace by solid-state fermentation with selected yeasts. LWT – Food. Sci. Technol. 2015;64(2):1342–1353.

[110] Ricci A, Cirlini M, Guido A, et al.. From By-product to resource: fermented apple pomace as beer flavoring. Foods. 2019,8(8):8. DOI:10.3390/foods8080309. PMC6723389 31374955

[111] Rebocho AT, Pereira JR, Neves LA, et al. Preparation and characterization of films based on a natural P(3HB)/mcl-PHA blend obtained through the co-culture of Cupriavidus Necator and Pseudomonas Citronellolis in Apple Pulp Waste. Bioengineering. 2020;7(2):32. 10.3390/bioengineering7020034 PMC7356164 32260526

[112] Bakshi PS, Selvakumar D, Kadirvelu K, et al.. Chitosan as an environment friendly biomaterial – a review on recent modifications and applications. Int J Biol Macromol. 2020;150:1072–1083. 31739057 10.1016/j.ijbiomac.2019.10.113

[113] Riaz A, Lagnika C, Abdin M, et al.. Preparation and characterization of Chitosan/Gelatin-Based Active Food Packaging Films Containing Apple Peel Nanoparticles. J Polym Environ. 2020;28(2):411–420.

[114] Vendruscolo F, Ninow JL. Apple pomace as a substrate for fungal chitosan production in an airlift bioreactor. Biocatal. Agric. Biotechnol. 2014;3(4):338–342.

[115] Torquati B, Marino D, Venanzi S, et al.. Using tree crop pruning residues for energy purposes: A spatial analysis and an evaluation of the economic and environmental sustainability. Biomass. Bioenerg. 2016;95:124–131.

[116] Frackowiak P, Adamczyk F, Wachalski G, et al.. A prototype machine for harvesting and baling of pruning residues in orchards: first test on apple orchard (MALUS MILL.) in Poland. J. Res. Appl. Agr. Engineering. 2016:61(3).

[117] Molinuevo-Salces B, González-Fernández C, Gómez X, et al.. Vegetable processing wastes addition to improve swine manure anaerobic digestion: evaluation in terms of methane yield and SEM characterization. Appl Energy. 2012;91(1):36–42.

[118] John J, Kaimal K, Smith ML, et al.. Advances in upstream and downstream strategies of pectinase bioprocessing: A review. Int J Biol Macromol. 2020;162:1086–1099. 32599230 10.1016/j.ijbiomac.2020.06.224

[119] Lyu F, Luiz SF, Azeredo D, et al.. Apple Pomace as a Functional and Healthy Ingredient in Food Products: A Review. Processes. 2020;8(3):319.

